# A therapeutic challenge relating to the association of orthostatic hypotension and supine hypertension in a patient with cardiac autonomic neuropathy: a case report

**DOI:** 10.1186/s13256-024-04346-0

**Published:** 2024-02-20

**Authors:** Jama Dounia, Haless Kamal, Selmaoui Marouane, Haboub Meryem, Habbal Rachida, Drighil Abdenasser, Azzouzi Leila

**Affiliations:** Service of Cardiology, CHUN Ibn Rochd: Centre Hospitalier Universitaire Ibn Rochd, Casablanca, Morocco

**Keywords:** Cardiac autonomic neuropathy, Autonomic nervous system, Orthostatic hypotension, Supine hypertension, Case report

## Abstract

**Background:**

Cardiac autonomic neuropathy is a highly prevalent pathology in the diabetic population, and is the leading cause of death in this population. Orthostatic hypotension is the main clinical manifestation of the disease. In some patients, this orthostatic hypotension is associated with supine hypertension, posing a therapeutic challenge since treatment of one entity may aggravate the other. The challenge is to manage each of these two hemodynamic opposites without exposing the patient to a life-threatening risk of severe hypotension or hypertension.

**Case presentation:**

We report a case of a 62-year-old ethnic Moroccan woman who has cardiovascular risk factors such as type 2 diabetes, arterial hypertension, and dyslipidemia. The patient’s symptoms included dizziness, tremors, morning sickness, palpitations, and intolerance to exertion. Given her symptomatology, the patient benefited from an exploration of the autonomic nervous system through cardiovascular reactivity tests (Ewing tests), which confirmed the diagnosis of cardiac autonomic neuropathy. In addition to orthostatic hypotension, our patient had supine arterial hypertension, complicating management. To treat orthostatic hypotension, we advised the patient to avoid the supine position during the day, to raise the head of the bed during the night, and to have a sufficient fluid intake, with a gradual transition from decubitus to orthostatism and venous restraint of the lower limbs. Supine hypertension was treated with transdermal nitrates placed at bedtime and removed 1 hour before getting up. One week after the introduction of treatment, the patient reported a clear regression of functional symptoms, with an improvement in her quality of life. Improvement in symptomatology was maintained during quarterly follow-up consultations.

**Conclusions:**

Cardiac autonomic neuropathy is a very common pathology in diabetic patients. It is a serious condition with a life-threatening prognosis. Its management must be individualized according to the symptomatology and profile of each patient. The treatment of patients with orthostatic hypotension and supine hypertension requires special attention to ensure that each entity is treated without aggravating the other.

## Background

Cardiac autonomic neuropathy (CAN) is the most common autonomic nervous system abnormality in diabetes. It corresponds to an alteration of the autonomic nerve fibers of the heart and blood vessels, leading to a dysregulation of the sympathovagal balance, resulting in abnormalities in the control of heart rate and blood pressure.

The prevalence of CAN confirmed in people with type 1 and type 2 diabetes is about 20%; this prevalence can increase with age and duration of diabetes and reach up to 65% [1]. CAN is often latent but severe, linked to excessive mortality related to the occurrence of cardiovascular complications including silent myocardial infarction, permanent tachycardia with shortened diastole and reduced myocardial viability, QT interval prolongation with risk of arrhythmia, and sudden death [2].

Despite these deadly consequences, CAN remains poorly studied on a practical level, and its detection, quantification, and treatment remain insufficient. The reduction of heart rate variability is one of the first signs of CAN but is generally unknown unless it is specifically sought in autonomic nervous system tests [3].

CAN is most often revealed by orthostatic hypotension (OH) defined by a decrease in systolic blood pressure of more than 20 mmHg and/or diastolic blood pressure of more than 10 mmHg within 3 minutes of decubitus [4].

In some patients, this orthostatic hypotension can coexist with supine hypertension, presenting a therapeutic challenge with the need to manage each of these two components without aggravating the other.

## Case presentation

We report a case of a 62-year-old ethnic Moroccan woman, who has cardiovascular risk factors such as type 2 diabetes, arterial hypertension, and dyslipidemia. The patient presented with a symptomatology made of vertigo and tremor, associated with a malaise when getting up in the morning. Her symptomatology was aggravated in warm atmospheres, with a feeling of well-being in cold climates. In addition, the patient presented palpitations with intolerance to the effort.

The clinical examination found a blood pressure of 159/101 mmHg in the right arm and 157/99 mmHg in the left arm, and a heart rate of 94 beats/minute. The cardiovascular examination was without special features, and the patient’s echocardiography was normal. The electrocardiogram showed a regular sinus rhythm at 94 beats/minute, and a fixed PR (PR is time between onset of atrial activation (P wave) and onset of ventricular myocardial activation (onset of QRS complex)) at 0.18 seconds without secondary repolarization disorders.

Given her symptomatology, the patient benefited from an exploration of the autonomic nervous system through cardiovascular reactivity tests (Ewing tests).

During this exploration, the patient was first placed in a calm atmosphere, in dorsal decubitus position on an examination table. Blood pressure and heart rate was measured every 5 minutes for 30 minutes, then the patient performed five tests of the autonomic nervous system, interspersed with periods of rest.

### The first test: deep breathing test

During this test, the patient performed six deep breaths for 1 minute. The patient’s calculated vagal response was 7% [normal (N): 30%], indicating a vagal deficiency.

### The second test: hand grip test

This test is a manual contraction effort performed to determine changes in blood pressure and heart rate at the static effort. In the patient, the vagal response was 6.5% (N: 10%), indicating a vagal deficiency

The peripheral sympathetic alpha response was 11% (N: 10%), reflecting peripheral sympathetic alpha hyperactivity.

### The third test: hyperventilation

This test corresponds to the patient performing shallow breathing for 15 seconds. The heart rate increased from 91 beats/minute to 97 beats/minute during this test. Blood pressure decreased from 171/100 mmHg to 144/86 mmHg.

### The fourth test: the mental stress test

This test consists of successive mental calculations leading to an increase in heart rate and blood pressure by increasing central sympathetic activity. The central alpha sympathetic response in the patient was 22% (N: 10%), indicating alpha sympathetic hyperactivity.

### The fifth test: orthostatic test

This test consisted of measuring the variation in heart rate and blood pressure in response to active lifting for at least 5 seconds after a period of rest in a supine position.

The patient’s blood pressure decreased from 159/99 mmHg to 128/80 mmHg, indicating the existence of orthostatic hypotension.

**Fig. 1:**
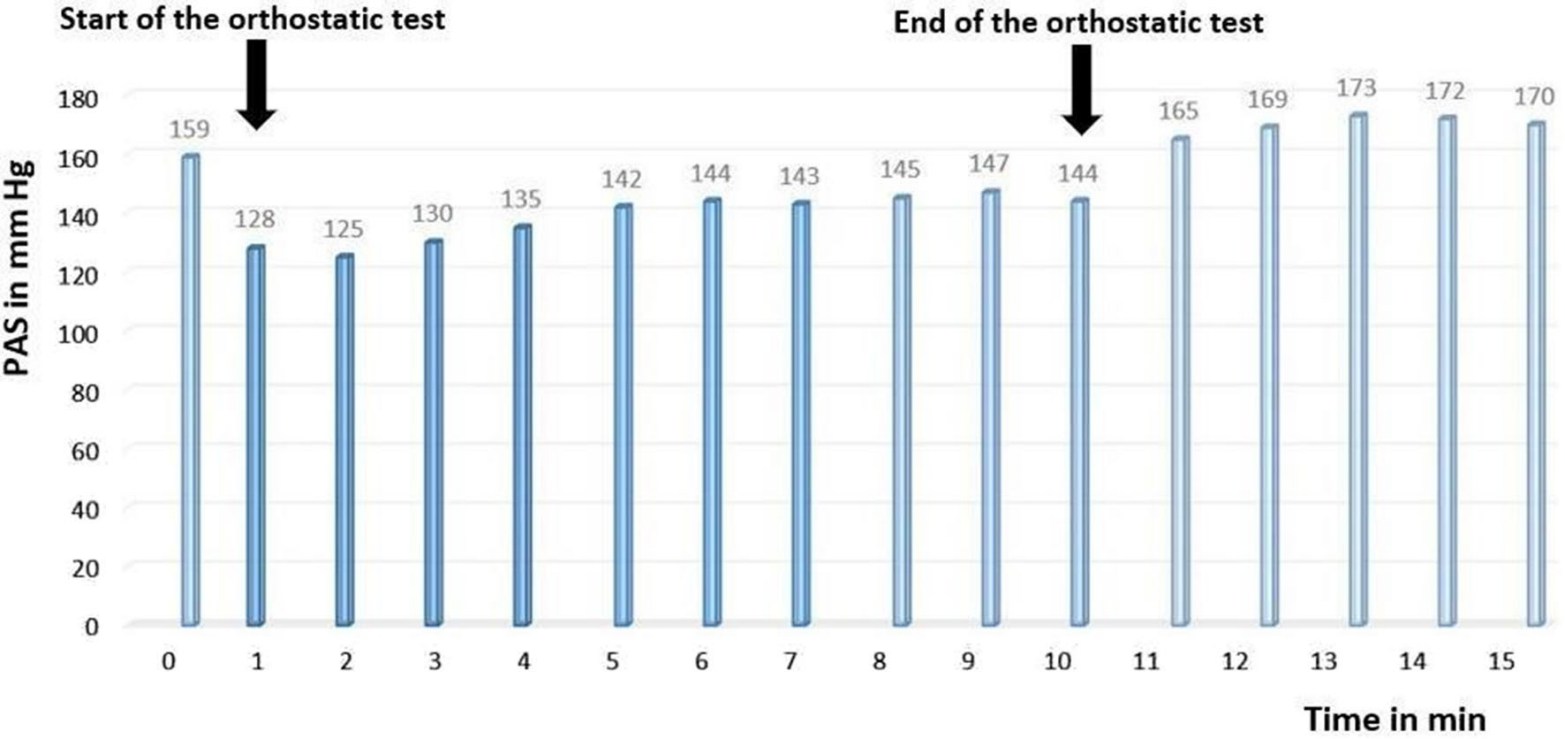
Evolution of systolic blood pressure during the orthostatic test showing orthostatic hypotension associated with supine hypertension

After the end of the orthostatic test, the shift to the supine position was associated with an increase in blood pressure to 173/110 mmHg in relation to supine hypertension (Fig. 1).

Table 1 summarizes the cardiovascular autonomic profile obtained from the cardiovascular reactivity tests (Ewing test):

**Table 1:** Autonomic profile obtained from cardiovascular reactivity tests

Test	Values
Vagal response to deep breathing	7% (N: 30%)
Peripheral alpha sympathetic	11% (N: 10%)
Central alpha sympathetic	22% (N: 10%)
Central beta sympathetic	9% (N: 10%)
Vagal response to orthostatic test	4% (N: 10%)

We note high blood pressure and heart rate in the basic state, a vagus deficiency in deep breathing and orthostatic test, sympathetic central and peripheral hyperactivity, and orthostatic hypotension associated with supine high blood pressure.

This autonomic profile is in favor of an autonomic cardiac neuropathy syndrome combining vagal deficiency and sympathetic hyperactivity.

## Discussion

CAN is a peripheral vegetative neuropathy, predominantly in small-caliber nerve fibers with little or no myelin. Representing one of the most frequent complications of diabetes, its prevalence increases with the duration of its evolution and the glycemic imbalance [5]. CAN induces an increase in cardiovascular morbidity and mortality; its symptomatology most often consists of orthostatic or postprandial hypotension, permanent tachycardia, silent myocardial ischemia, and sudden death secondary to severe cardiac rhythm disorders. Only 24% of patients present signs suggestive of dysautonomia, hence the interest to systematically search for CAN in diabetic patients [5].

Its diagnosis is based on clinical tests of the autonomic nervous system showing a vagal deficiency, which is often associated with sympathetic hyperactivity.

The resting heart rate is approximately 10 beats/minute higher in diabetic subjects with CAN than in those without CAN. This resting tachycardia can be explained by parasympathetic damage, which generally precedes orthosympathetic damage. Dynamic tests also show a decrease in heart rate variability in these patients, and therefore an abnormality in the adaptation of the heart rate to circadian variations, stress, and exercise, which is the cause of the exercise intolerance frequently experienced by patients with CAN [6–8].

Orthostatic hypotension is a later sign that can be isolated or associated with the occurrence of a sensation of fatigue, dizziness, lipothymia, or even syncope. This symptomatology is due to an abnormality in the functioning of the baroreflex arc with a defect in the ascent of the heart rate and myocardial ionotropic during the transition to the standing position [4].

In some patients, this orthostatic hypotension may coexist with supine hypertension, which poses a therapeutic problem because the treatment of one entity may aggravate the other.

Management of combined orthostatic hypotension and supine hypertension: in most patients with orthostatic hypotension with supine hypertension, there are good reasons to prioritize the treatment of orthostatic hypotension over supine hypertension [9, 10].

Symptomatic orthostatic hypotension is accompanied by various debilitating symptoms, including postural dizziness, syncope, fatigue, weakness, and visual disturbances. All of these symptoms can contribute to an increased frequency of falls with numerous complications and risk of death [11].

Fludrocortisone should be avoided when treating OH in patients with an association of OH with supine hypertension [11]. The use of midodrine, which is a peripheral and selective alpha adrenergic receptor agonist that acts by increasing arterial resistance and venous return, carries a risk of significant supine hypertension, so it is recommended that patients do not take midodrine within 5 hours before bedtime [9–12] and do not rest or sleep in the supine position; on the contrary, decubitus should always be assumed in the head-up position [13].

Expert recommendations for the management of supine hypertension in the setting of orthostatic hypotension suggest that supine hypertension requires intervention if systolic blood pressure exceeds the range of 160–180 mmHg. Many medications used to treat orthostatic hypotension can cause or exacerbate supine hypertension, given that orthostatic hypotension and supine hypertension are hemodynamic opposites, amelioration of one may worsen the other [11].

All patients with OH and supine hypertension should be advised to avoid the supine position during the day and elevate the head of the bed as tolerated at night. Clinicians should manage patients with significant supine hypertension with short-acting antihypertensive drugs such as captopril, clonidine, hydralazine, and losartan administered at bedtime and not during the day to avoid the worsening of OH during the day [14].

The use of transdermal nitrates is the treatment of choice for supine hypertension [15]. It is recommended that the nitrate patch be applied at bedtime and removed 1–2 hours before getting up [11].

In our patient, we indicated hygienic and dietetic measures to treat orthostatic hypotension, in particular sufficient fluid intake, a gradual transition from decubitus to orthostasis, venous restraint of the lower limbs, and elevation of the head of the bed by 5° to 10°. For the treatment of supine hypertension, we opted for transdermal nitrates placed at bedtime and removed 1 hour before getting up.

One week after the introduction of treatment, the patient reported a clear regression of functional symptoms, with an improvement in her quality of life. Improvement in symptomatology was maintained during quarterly follow-up consultations.

## Conclusions

Cardiac autonomic neuropathy is a highly responsive pathology in diabetic patients occurring in the medium or long term during the course of diabetes, corresponding to an alteration of the cardiovascular system’s sympathetic and parasympathetic autonomic control.

The alteration of the functioning of the baroreflex arc during CAN is responsible for the appearance of orthostatic hypotension, which is added in 50% of the patients to supine hypertension, exposing a therapeutic problem because the treatment of one entity can aggravate the other.

In these patients, orthostatic hypotension management consists of adopting hygienic and dietetic measures (abundant fluid intake, a progressive transition from decubitus to orthostatism, venous restraint of the lower limbs, and so on). Avoidance of diuretics and long-acting antihypertensive drugs is necessary. Midodrine should not be taken within 5 hours before bedtime to avoid the aggravation of supine hypertension.

As for the management of supine hypertension, short-acting antihypertensive drugs such as nitrates, captopril, losartan, and clonidine should be administered at bedtime and not during the day to avoid the worsening of OH.

## Data Availability

Non-applicable.

## Abbreviations

CAN: Cardiac autonomic neuropathy
OH: Orthostatic hypotension
N: Normal
